# Protease Produced by Endophytic Fungi: A Systematic Review

**DOI:** 10.3390/molecules26227062

**Published:** 2021-11-22

**Authors:** Victor Hugo Souto Bezerra, Samuel Leite Cardoso, Yris Fonseca-Bazzo, Dâmaris Silveira, Pérola Oliveira Magalhães, Paula Monteiro Souza

**Affiliations:** Natural Products Laboratory, Campus Darcy Ribeiro, School of Health Sciences, University of Brasilia, Brasília 70910-900, DF, Brazil; victor.souto@aluno.unb.br (V.H.S.B.); samuel.leite@aluno.unb.br (S.L.C.); yrisfonseca@unb.br (Y.F.-B.); damaris@unb.br (D.S.); perolamagalhaes@unb.br (P.O.M.)

**Keywords:** protease, endophytic fungi, systematic review

## Abstract

The purpose of this systematic review was to identify the available literature of production, purification, and characterization of proteases by endophytic fungi. There are few complete studies that entirely exhibit the production, characterization, and purification of proteases from endophytic fungi. This study followed the PRISMA, and the search was conducted on five databases: PubMed, PMC, Science Direct, Scopus Articles, and Web of Science up until 18 May 2021, with no time or language restrictions. The methodology of the selected studies was evaluated using GRADE. Protease production, optimization, purification, and characterization were the main evaluated outcomes. Of the 5540 initially gathered studies, 15 met the inclusion criteria after a two-step selection process. Only two studies optimized the protease production using statistical design and two reported enzyme purification and characterization. The genus *Penicillium* and *Aspergillus* were the most cited among the eleven different genera of endophytic fungi evaluated in the selected articles. Six studies proved the ability of some endophytic fungi to produce fibrinolytic proteases, demonstrating that endophytic fungi can be exploited for the further production of agents used in thrombolytic therapy. However, further characterization and physicochemical studies are required to evaluate the real potential of endophytic fungi as sources of industrial enzymes.

## 1. Introduction

The use of chemicals worldwide in different industries has increased the demand for industrial enzymes, mainly due to the need for new sustainable industrial processes that do not affect people’s health. Furthermore, enzymes also help preserve an unpolluted environment through their use in waste management, where they are used for effluent treatment and detoxification, renewable energy resources, bioindicators for pollution, and biosensors [1]. With the advancement in technology, a large part of enzymes of industrial and pharmaceutical interest began to be produced on a large scale [2,3]. Proteases are enzymes whose catalytic function is to hydrolyze peptide bonds of proteins into amino acids and peptides. They are part of a large group of enzymes belonging to the class of hydrolase enzymes [4,5].

Currently, proteases are used in several industries such as the pharmaceutical industry, the leather industry, the detergent, and food industries [6,7]. In the food industry, proteases have several functions and are used in different sectors such as cheese-making, bakery, and meat production. The use of proteolytic enzymes in the leather industry has been replacing chemical agents that are toxic and dangerous to the environment. In medical applications, protease can be used to treat a variety of diseases such as cancer, inflammatory diseases, glaucoma, etc. The use of proteases in the detergent industry is responsible for approximately 20% of the market for commercial enzymes [3,8,9].

Proteases can be produced from plants, animals, and microorganisms. The production of proteases by microorganisms has grown in recent years due to the wide variety of enzymes they can produce. They represent about two-thirds of the world’s production of proteases [8]. The production of proteases by fungi has grown in recent years due to the wide variety of enzymes produced and the easy separation of mycelia from the culture media when compared to bacteria [9,10]. In addition, fungi are generally considered recognized as GRAS (generally regarded as safe), and the proteases produced are mostly extracellular, which facilitates the removal of the enzyme from the fermentation broth [11]. Currently, fungi are responsible for 60% of the enzymes used in the most diverse industries [12]. A wide variety of fungi are known to be great sources of active and functional proteases. The most used strains for the production of industrial proteases are of the genus *Aspergillus* and *Trichoderma* [13].

**Figure 1:**
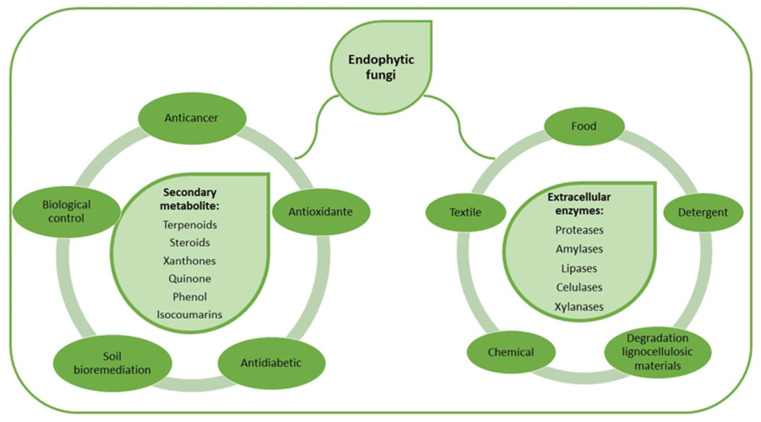
Biotechnology application of secondary metabolites and extracellular enzymes produced from endophytic fungi.

The term “endophytic” is used to define all the organisms that inhabit the internal tissues of their living hosts during some period of their life cycle [14,15], therefore, endophytic fungi are microorganisms that inhabit the different internal tissues of plants without causing any apparent damage to the host plant [16]. Endophytic fungi have proven to be a source of great potential to produce secondary metabolites and several extracellular enzymes such as amylases, lipases, and proteases as part of their defense mechanisms against other organisms and to obtain the necessary nutrients for their development. The major secondary metabolites and extracellular enzymes synthesized from endophytic fungi, and their potential applications are represented in Figure 1. In recent years, several studies have been carried out to identify endophytic fungi capable of producing proteases with potential industrial or pharmaceutical applications [15,17,18,19].

Nowadays, there is a worldwide movement toward prospecting new means of the production of active principles obtained by biotechnological processes. In this context, endophytic fungi have been explored in their biological diversity as new sources of enzymes. Therefore, the isolation and identification of endophytic fungi, the recognition of potential protease producers, and the study of the characteristics of these proteases have become important due to the great biotechnological potential of endophytic fungi as sources of novel proteases that can be useful for specialized industries. Therefore, this review aimed to evaluate and identify the current state of production, characterization, and purification of proteases by endophytic fungi in the available literature.

## 2. Results

### 2.1. Study Selection

A total of 6028 articles were found after applying the search procedures initially established across the five electronic databases. In the PMC databases were found 2731 articles, 2176 in the Scopus databases, 733 in Science Direct databases, 218 in Web of Science, and 170 in PubMed. After the search process, duplicates were removed, leaving 5261 references. An evaluation of the titles and abstracts of the articles was carried out, thereby excluding 5191 references and retaining 70 references. Any article was identified using the Google Scholar platform. Therefore, supported by the inclusion and exclusion criteria, a review of the full texts was completed, and 15 articles were selected for this review [20,21,22,23,24,25,26,27,28,29,30,31,32,33,34]. This process led to the exclusion of 55 papers (Appendix A). A flow chart detailing the process of identification, inclusion, and exclusion of studies is shown in Appendix B.

### 2.2. Study Characteristics

A summary of the descriptive characteristics of the included studies is provided in Table 1. The selected articles were carried out in eight different countries. One each from Tunisia [20], Malaysia [30], Australia [33], and the United States [25]; two from Egypt [22,34], Brazil [23,26], and China [24,32]; and five from India [21,27,28,29,31]. The articles were published between 1994 and 2021 and were written in English. All articles evaluated the production of proteases by endophytic fungi, and nine of the articles performed purification processes and characterization of the proteases produced as described in Table 2 [22,24,25,27,28,29,30,32,34].

The N-terminal amino acid sequence of the purified protease was only performed by three articles. Wu et al. [32] used the automated Edman method to determine the N-terminal sequence of a fibrinolytic protease. The N-terminal sequence of the protease produced by *Fusarium* sp. (QASSGTPATIRVLVV) appeared to differ strongly from other reported fibrinolytic proteases. The N-terminal sequence of a fibrinolytic protease produced by *X. curta* was also determined by the automated Edman degradation method. The N-terminal sequence (SNGPLPGGVVWAG) showed differences from previously reported fibrinolytic enzymes from fungi.

### 2.3. Synthesis of Results

#### 2.3.1. Microorganism

Among the 15 articles selected for this review, eleven different genera of endophytic fungi were reported as protease producers: *Penicillium bilaiae* [20], *Talaromyces flavus* [21]; *Mortierella hyalina* [21]; *Paecilomyces variabilis* [21]; *Penicillium* sp. [21,26], *Aspergillus ochraceus* [22,34], *Aspergillus niger* [23], *Verticillium* sp. [24], *Acremonium typhinum* [25], *Aspergillus* sp. [26], *Lasiodiplodia pseudotheobromae* [27], *Xylaria curta* [28,29], *Penicilium citrinum* [30], *Fusarium* sp. [30,32], and *Alternaria alternata* [31,33]. In eleven articles, the molecular identification of the fungus involved was mentioned, which species were identified based on ITS-rDNA [22,25,29,31,34,35], 18S rRNA sequence analysis [22,34], PCR amplification of the β-tubulin gene [21], based on the 28s rDNA region [28,29] and identified on the basis of cultural characteristics, color, and morphology of fruiting bodies and spores [24,31].

#### 2.3.2. Optimization of Protease Production

Of the 15 studies evaluated, four carried out processes to optimize the production of proteases. Mefteh et al. [20] used a response surface methodology (RSM) tool and Plackett–Burman design to optimize the production of protease by the endophytic fungus *P. bilaiae*. Elgammal et al. [22] and Rajput et al. [31] adopted a procedure for the optimization of protease production by fungi *A. ochraceus* and *A. alternata*, respectively, evaluating different parameters such as incubation time, pH, temperature, carbon and nitrogen sources independently while keeping the other parameters constant. Zaferanloo et al. [33] applied a factorial experiment based on a randomized complete design to optimization of protease production by *A. alternata*.

#### 2.3.3. Growth Conditions

Culture medium with different carbon and nitrogen sources were used to produce protease from endophytic fungi. Three studies used Czapek Dox broth or this medium supplemented with other nitrogen and carbon sources for the cultivation of endophytic fungi to induce the protease production [29,30,35]. The culture medium used by Li et al. [24] containing 3.0% sucrose, 0.3% NaNO_3_, 0.1% K_2_HPO_4_, 0.1% yeast extract, 0.05% KCl, 0.05% MgSO_4_·7H_2_O, and 0.001% FeSO_4_ had a protease activity of 3.775 UI/mg. The activity found by Meshram and Saxena [27] by the cultivation of the fungus *L. pseudotheobroma* in Czapek Dox broth, which is composed of sucrose, NaNO_3_, MgSO_4_, KCl, and FeSO_4_ was 3.56 UI/mg. The presence of an organic nitrogen source (yeast extract) may have induced a greater production of protease by the fungus *Verticillium* sp. [24]. Among the fifteen studies evaluated, twelve used submerged fermentation (SmF), a temperature ranging from 23 °C to 35 °C, and agitation from 110 to 220 rpm for the endophytic fungus cultivation [20,21,22,23,24,26,27,28,31,32,33,34]. Only one study used solid-state fermentation (SSF) to produce protease [29]. Two other studies did not mention the cultivation method used [25,30].

#### 2.3.4. Assay for Protease Activity

Proteolytic activity can be measured by different methods using several substrates. In this systematic review, four selected studies used casein to quantify the enzyme activity [22,25,33,34], three articles performed a protease assay using azocasein [24,28,36], and one article used azoalbumin [25]. Noor et al. [30] estimated the activity using a kit that quantifies protease with a fluorescein thiocarbamoyl-casein derivative (FTC-casein). Zaferanloo et al. [33] applied a QuantiCleaveTM Protease Assay Kit (Thermo-Scientific, Waltham, MA, USA) that uses succinylated casein as a substrate. Four articles performed specific assays for fibrinolytic enzymes, three of which determined the activity via fibrin plate assay [27,28,29]. Li et al. [24] performed a method slightly modified from Qiuling et al. [35].

#### 2.3.5. Enzyme Characterization

The effect of pH and temperature were reported by ten studies [20,22,24,25,28,29,30,32,33,34], and maximum protease activities were observed at value pH range from 6 to 10 and temperature ranged between 25 °C and 60 °C. Six proteases showed an optimum pH of 8 [22,28,29,30,32,34]. Three proteases produced by *Fusarium* sp. [30], *A. alternata* [33], and *P. bilaiae* [20] had the highest activity at neutral pH (6–7). Alkaline proteases produced by *Verticillium* sp. [24] and *A. typhinum* [25] showed an activity peak at pH 9–11. Proteases produced by *A. ochraceus* [22,34] and *X. curta* [28,29] showed an optimum temperature of 50 and 35 °C, respectively.

Seven of the 15 selected articles evaluated the effect of inhibitors on proteases [22,26,27,30,31,34,36]. The protease produced by *P. bilaiae* [20] was completely inhibited by the inhibitor phenylmethylsulfonyl fluoride (PMSF), a serine protease inhibitor. Li et al. [24] found that the protease produced by *Verticillium* sp. could be inhibited by PMSF and dithiothreitol (DTT). Protease produced by *A. ochraceus* was also inhibited by PMSF, however, it was stimulated by DTT and β-mercaptoethanol [34]. This enzyme was classified as a thiol-dependent-serine protease. Lindstrom and Belanger [25] demonstrated that the protease produced by *A. typhinum* was partially inhibited by PMSF and dichloroisocoumarin (DCI), also a serine protease inhibitor. The proteases produced by *X. curta* in two studies were completely suppressed by metalloprotease inhibitors such as ethylenediaminetetraacetic acid (EDTA) and ethylene-bis(oxyethylenenitrilo)tetraacetic acid (EGTA) [28,29]. Wu et al. [32] found that the protease produced by *Fusarium* sp. could be inhibited by PMSF and EDTA.

Eight studies determined the molecular weight of proteases [24,25,27,28,29,30,32,34]. The identified size of proteases found in the different studies ranged from 28 to 80 kDa., and among them, six ranging in size from 28 to 34 kDa [24,25,28,29,30,32]. The highest protease produced by *Verticillium* sp. activity found by Li et al. [24] had a molecular weight of 31 kDa.

Meshram et al. [29] was the only study that determined the kinetic parameters of the studied protease produced by the endophytic fungus *X. curta*. The enzyme *K_m_* and *V_max_* for the azocasein substrate were 326 µM and 0.13 µM min^−1^, respectively.

The isoelectric point was determined by only one article. Wu et al. [32] isolated and purified a fibrinolytic protease from *Fusarium* sp., and the protein presented an isoelectric point of 8.1.

One study determined the thermal stability of the protease [20]. A protease produced by fungus *P. bilaiae* was evaluated for its thermostability. After 10 min at 70 °C, there was no activity. The enzyme remained stable after 20 min at 30 °C.

#### 2.3.6. Purification

Of the 15 articles selected, eight performed partial or complete purification processes of the protease [22,24,25,27,28,29,30,32]. Two studies performed a partial purification of the protease produced by *A. ochraceus* [22] and *L. pseudotheobromae* [27] using ethanol and ammonium sulfate precipitation, respectively, followed by dialysis. In another study, this same protease from *A. ochraceus* was completely purified using precipitation by ammonium sulfate followed by gel filtration and ion exchange chromatography (Sephacryl S-200, DEAE-Sepharose, and CM-Sepharose columns, respectively) [34]. Lindstrom and Belanger [25] purified a protease produced by *A. typhinum* using ultrafiltration methods (Centripep-30) followed by passage in a phenylboronate column and finally, precipitation with methanol. Six studies used more complete purification methods with initial precipitation with ammonium sulfate followed by chromatographic processes using ion exchange columns and size exclusion columns. Li et al. [24] purified a fibrinolytic protease produced by *Verticillium* sp. using a combination of sequential chromatography composed of DEAE-52, Sephadex G-75, and hydrophobic columns. A fibrinolytic enzyme produced by *X. curta* was purified using gel filtration chromatography with a Sephacryl S-300 column [28,29]. Noor et al. [30] used a combination of fast protein liquid chromatography (FPLC) equipped with a Hi-Prep 26/10 Desalting Column and Hi-Trap Benzamidine FF/Hi-Trap IEX Selection Kit to purify proteases produced by *Fusarium* sp and *P. citrinum*. Wu et al. [32] purified a fibrinolytic protease produced by *Fusarium* sp. by employing two steps: passing through the MonoQ column and Superdex 75 column.

### 2.4. Risk of Bias

The articles selected in this study were evaluated using the GRADE tool, as seen in Table 3. Two studies were graded as very low quality and three as low quality. Bhagobaty and Joshi [21] was classified with serious study limitations and publication bias because it used methods with a high degree of interference for the quantification of proteases. Lindstrom and Belanger [25] and Rajput et al. [31] were scored as serious limitation because they did not show the sample size or the assay was not performed in at least triplicate. Noor et al. [30] did not use a specific substrate to assess the fibrinolytic activity of the produced protease by the endophytic fungus. Six studies were rated as moderate quality and three as high quality. Of the 15 articles evaluated, nine were scored as inconsistent because they did not present statistical analysis of the data obtained or did not mention whether tests were performed in triplicate.

## 3. Discussion

In recent years, endophytic fungi have been shown to be a source of great potential to produce bioactive compounds with promising applications in agriculture, the environment, the pharmaceutical industry, and the food industry. When compared to other endophytic microorganisms, endophytic fungi produce a wider range of active compounds and these compounds have a broad biological activity [18]. Eleven different genera of endophytic fungi were identified in the evaluated articles (*Penicillium, Talaromyces, Mortierella, Paecilomyces, Aspergillus, Verticillium, Acremonium, Lasiodiplodia, Xylaria, Fusarium,* and *Alternaria*). Meshram et al. [28] reported for the first time the production of a fibrinolytic protease by *Xylaria* species. In this review, only studies that presented quantitative data to produce proteases were selected. However, several other studies have qualitatively evaluated endophytic fungi as potential producers of proteases [36,37,38,39].

Nowadays, the analysis of variables through statistical methodologies are widely used to optimize the enzymatic production of several microorganisms. They are quick and easy methodologies and are quite reliable [40]. However, few studies using statistical techniques have been conducted for protease optimization by endophytic fungi. Only two studies used statistical methodologies to optimize the production of proteases by endophytic fungi. Mefteh et al. [20] used two methods, Plackett–Burman design and Box Behnken design, experimental designs for response surface methodology. Zaferanloo et al. [33] used a factorial experiment based on a randomized complete design as a statistical tool for optimizing protease production. The use of statistical techniques is a better tool for optimizing enzyme production than the traditional one-variable-at-a-time method, as they allow not only to assess the individual influence that each factor exerts on enzyme production, but also the interaction between them.

The production of proteases by microorganisms is greatly influenced by the components present in the culture medium, especially carbon and nitrogen sources, metal ions, some physical factors (pH and temperature), incubation time, and inoculum size [10]. It is known that proteases are usually produced in the stationary phase of growth, and therefore, carbon and nitrogen sources exert regulatory effects on enzyme synthesis [41]. The way each fungus uses carbon and nitrogen sources is individual and depends on several factors, so there is no specific culture medium to produce proteases, as the ideal pH and temperature vary from fungus to fungus. Two studies used the Czapek Dox as culture medium, which contains sucrose as a carbon source and sodium nitrate as a nitrogen source [27,28]. Meshram et al. [29] was the only study that used the solid-state fermentation technique and evaluated the influence of different agro-industrial residues (rice chaff, wheat bran, eggshell, orange peel, and banana peel) on enzyme production by the fungus *X. curta*. Four articles evaluated the effect of different carbon and nitrogen sources on protease production. Mefteh et al. [20] found mannose and malt extract as the best carbon sources in the production of protease by the fungus *P. bilaiae*. Elgammal et al. [22] showed that dextrin was the best source of carbon and peptone was the best source of nitrogen to produce protease by the fungus *A. ochraceus*. Two studies that evaluated the best sources of carbon and nitrogen to produce protease by the fungus *A. alternata* found that glucose and soybean were the best source of carbon and yeast extract was the best source of nitrogen [31,33].

In addition to carbon and nitrogen sources, physical factors also influence the induction or repression of protease production such as initial pH, temperature speed agitation, and inoculum size. Among the physical parameters, pH and temperature are important regulators of enzyme production and the stability of substrates in the culture medium because it can affect the chemical structure of enzymes, causing their denaturation and loss of catalytic activity [22]. Three studies indicated that some physical parameter negatively or positively influenced the production of proteases. Mefteh et al. [20] evaluated the influence of the initial temperature and pH of the medium on the production of protease from the endophytic fungus *P. bilaiae*. Elgammal et al. [22] performed tests to measure the effect of initial pH, temperature, inoculum level, and agitation on protease production by the endophytic fungus *A. ochraceus*. Meshram et al. [29] analyzed how the parameters of temperature, incubation time, and particle size influenced the production of proteases from the endophytic fungus *X. curta*.

The discovery of new sources that produce proteases such as endophytic fungi could be a good strategy to produce these enzymes at an industrial level. The production of a wide range of proteases with different thermodynamic characteristics implies the wide applicability of these enzymes in the food, pharmaceutical, textile, paper, and sanitizing products industries.

Alkaline proteases could be used as bio-additive compounds in the textile and food industries and to increase cleaning power [36,42,43]. In this review, Li et al. [24] and Lindstrom and Belanger [25] found fungal alkaline proteases of pH 9, 10, and 11, respectively, however, there was no assay for the application of these enzymes. The works published by Meshram et al. [28] and [29], Noor et al. [30], and Wu et al. [32] demonstrated the fibrinolytic protease application with optimal pH 8.

The kinetic parameters as well as the thermal stability and the isoelectric point have only been presented in a few studies [20,29]. The enzymatic characterization is important to evaluate the economic and industrial application of these enzymes. It is of fundamental importance to understand the functioning and characteristics of each protease in order to apply them in processes of industrial magnitude [44].

The enzymatic activity must be evaluated by analyzing the substrate in the reaction, and the type of protease. Characterization in serine, cysteine, or metalloprotease can be performed by testing with inhibitors, as demonstrated by Li et al. [24], Mefteh et al. [20], Wu et al. [32], Meshram et al. [29], Lindstrom and Belanger [25], and Meshram et al. [28].

The analysis of the N-terminal sequence of proteins allows for a further comparison with proteins already studied in previous works. The N-terminal sequence analysis of the fibrinolytic protease found by Meshram et al. [29] allowed for the discovery of a bifunctional enzyme with no homology to those deposited fibrinolytic proteases in the databases. According to Luo et al. [45], the analysis of the N-terminal sequence of proteins allows for confirmation of the identity of the protein, thus providing additional information on mass and subunits.

The protein purification process, in general, involves a series of actions to isolate a specific protein present in a complex mixture and remove unwanted compounds. An ideal purification process should be carried out with the fewest possible steps to avoid loss of the desired protein and be a low-cost process. However, the processes to be used will depend on the final application of the enzyme. The first step in recovering extracellular proteases involves separating cell biomass from the fermentation broth. The next step involves concentrating the proteases, which can be conducted through filtration or precipitation methods. In the following steps, generally, procedures involving column chromatography are performed. Among the selected articles, two performed partial purification that involved enzyme precipitation processes with ethanol [22] and ammonium sulfate [27] followed by dialysis. Six studies applied ammonium sulfate precipitation methods followed by chromatographic processes using ion exchange chromatography, size exclusion chromatography [26,30,31,34,36], and affinity chromatography for enzyme purification [30]. One study used ultrafiltration methods (Centripep-30) followed by passage in a phenyl boronate column and finally, precipitation with methanol [25].

As mentioned, proteases are constantly used by the pharmaceutical industry to produce cosmetics and medicines. Recently, endophytic fungi have been used as precursors to produce proteases with potential fibrinolytic action that can play an important role in thrombolytic therapy. Among the articles in this review, six explored the potential of endophytic fungi to produce fibrinolytic proteases. The endophytic fungi *Verticillium* sp. [24], *L. pseudotheobromae* [27], *X. curta* [28,29], *Fusarium* sp. [30,32], and *P. citrinum* [30] were able to produce fibrinolytic proteases, with potential industrial application for the formulation of agents used in thrombolytic therapy.

## 4. Materials and Methods

This systematic review was conducted following the PRISMA (Preferred Reporting Items for Systematic Reviews and Meta-Analysis) Checklist [46]. The protocol was not registered because it is a systematic review of in vitro studies. This type of systematic review is not suitable for inclusion in the PROSPERO (International Prospective Register of Systematic Reviews).

### 4.1. Information Sources and Search Strategy

To conduct this systematic review, specific research strategies were carried out in five bibliographic databases (Appendix C): PubMed, PMC, Science Direct, Scopus Articles, and Web of Science. A separate search was carried out in the Google Scholar database in case any relevant study was not selected during the search in the five electronic databases. The search for articles in electronic databases was performed independently by two authors on the same day to ensure that the search was carried out correctly. The research included only scientific articles published before 10 September 2021 with no time or language restrictions. A reference manager software was used to remove duplicate references (EndNote, Thomson Reuters, Toronto, ON, Canada).

### 4.2. Study Selection

The selection of the articles was conducted in two stages. In stage one, the titles and abstracts of all articles were analyzed independently by two authors. This first review selected articles that seemed to meet the inclusion criteria based on the title and abstract. When any divergence appeared between the two initial authors, a third author was consulted to resolve it. Studies that did not have any inclusion criteria or that were not related to the topic of this review were excluded. In stage two, two authors read the entire text of the remaining articles and excluded those that did not meet the inclusion criteria. Finally, three authors reviewed the remaining articles and selected the articles evaluated in this review.

### 4.3. Eligibility Criteria

#### 4.3.1. Inclusion Criteria

For this review, articles were selected that showed the enzymatic activity of proteases produced by endophytic fungi of any species, evaluated the optimization of the production of proteases, and performed purification processes (complete or partial) or enzyme characterization (temperature, pH, isoelectric point, stability).

#### 4.3.2. Exclusion Criteria

Articles that presented any of the following items were excluded: (1) studies performed with non-endophytic fungal species; (2) papers with only screening qualitative studies or did not quantify the protease activity; (3) reviews, letters, personal opinions, book chapters, and conferences; (4) studies that did not mention the production of proteases by endophytic fungi; and (5) studies written in non-English language. For this review, only endophytic fungi isolated from organisms belonging to the kingdom Plantae were considered. Studies involving mycorrhizal fungi, sometimes classified as endophytic fungi, were disregarded as they were considered to be different species [47].

### 4.4. Data Collection Process and Data Items

Data collection from the selected articles was carried out by two authors independently. The third author was responsible for checking the collected data. Any disagreement was resolved after discussion between the three authors and mutual agreement. From the articles included in this review, the following data were collected: year of publication, author(s), country and site, fungus species, plant species from which the fungus was isolated, protease activity, growth condition, purification processes, and data from enzyme characterization.

### 4.5. Risk of Bias in Individual Studies

An assessment tool of the quality of evidence from studies was employed as the methodology in the evaluation of selected studies in this review [48]. The GRADE tool used was adapted for in vitro studies, since no other specific methodology for quality analysis has been developed so far. Two authors independently ranked each item according to its quality as ‘high’, ‘moderate’, ‘low’, or ‘very low’. Disagreements were resolved by a third author.

### 4.6. Risk of Bias in Individual Studies

Production, optimization, purification, and characterization of proteases from endophytic fungi were the main evaluated outcomes.

## 5. Limitations

After analyzing the articles included in this review, some points need to be considered. Among the selected articles, only two performed enzyme optimization processes. Only two had optimized enzyme production with the aid of statistical techniques, showing that statistical methodologies should be used more in optimization processes, since evaluating the effects of the physical and chemical parameters using the method of one variable at a time, in the view of the authors of this review, only evaluates the individual effects of these on the parameters in enzyme production and does not actually optimize production. The GRADE tool used was adapted for in vitro studies, since no other specific methodology for quality analysis has been developed thus far. Only four studies were rated as high quality, showing that most of the studies evaluated had some risk bias or were not able to fully answer the review questions defined in this systematic review.

## 6. Conclusions

This systematic review showed different species of endophytic fungi as excellent producers of proteases, with potential applications in different industrial segments. The genus *Penicillium* was the most cited among the eleven different genera of endophytic fungi evaluated in the selected articles, followed by *Aspergillus*, *Alternaria*, and *Xylaria*. It is known that the use of statistical methodologies is a better tool to optimize the growth conditions for enzyme production, however, only two used statistical methodologies to optimize the protease production. This demonstrates a lack of studies that use more effective techniques to improve the yield of proteases so that they can be produced on a large scale. Enzyme characterization is an important process that is performed to understand the functionalities and characteristics of a protease to assess its economic and industrial potential. Only two studies carried out characterization of the protease found. In the evaluated articles, different sources of carbon and nitrogen were used in the culture media. This infers that those endophytic fungi can produce proteases with a wide variety of carbon and nitrogen sources. Six studies proved the ability of some endophytic fungi to produce fibrinolytic proteases, demonstrating that endophytic fungi can be exploited as producers of fibrinolytic proteases for the further production of agents used in thrombolytic therapy. Therefore, the great potential of endophytic fungi as a source of proteases was observed with potential application in the pharmaceutical industry.

## Figures and Tables

**Table 1:** Summary of the descriptive characteristics of the included studies (N = 15).

Author	Country	Host Plant	Microorganisms	Fungal Identification	Growth Conditions				Enzyme Activity	Main Conclusions
					Ph	T (°C)	Agitation (rpm)	Time (days)		
Mefteh et al. [20]	Tunisia	*Phoenix* *dactylifera* L.	*Penicillium bilaiae*	ITS-rDNA	6.26	24.5	150	ND	1086.95 U/mL	Plackett–Burman design and RSM approaches were employed for optimization of culture and environment conditions and were shown to significantly enhance protease production.
Bhagobaty and Joshi [21]	India	*Potentilla fulgens*	*Talaromycesflavus*	β-tubulin	ND	25	120	5	34.9 U/h/mL	All the endophytic fungal isolates from medicinal plant showed production of protease. The production of extracellular enzymes was greater in the liquid medium in comparison to the plate-based assays.
Elgammal et al. [22]	Egypt	*Ruprechita salicifolia*	*Aspergillus ochraceus*	18S rRNA	8	35	150	ND	292 U/mL	The protease production increased by about 7.5-fold after applying the final optimized fermentation. The partial purification results showed that the highly recovered fraction was at 60% ethanol concentration.
El-Khonezy et. al. [34]	Egypt	*Ruprechita salicifolia*	*Aspergillus ochraceus*	18S rRNA	8	35	150	6	800.1 U/mL	The enzyme was characterized as thiol-dependent serine alkaline protease. Low-cost production medium using different waste sources was applied to produce the enzyme.
Galeano et al. [23]	Brazil	*Axonopus purpusii*	*Aspergillus niger*	ITS-rDNA	ND	30	110	7	12.01 U/mL	The ability of the fungus to produce proteases might reflect the fact that these fungi have potential as biocontrol agents.
Li et al. [24]	China	*Trachelospermum jasminoides*	*Verticillium* sp.	Morphology	ND	28	160	14	3775 U/mg	Verticase is a direct degrader of fibrin clot, most probably playing a negligible role in the conversion of plasminogen to plasmin. However, for protein-based medicines, special care must be taken for an early awareness of the toxicity.
Lindstrom and Belanger [25]	United States	*Poa ampla*	*Acremonium typhinum*	ND	ND	ND	ND	ND	27 U/mL	The regulated nature of proteinase At1 suggest that its function is important in the symbiotic interaction of fungus and plants.
Matias et al. [26]	Brazil	*Myrcia guianensis*	*Aspergillus* sp.	Morphology ^a^	5	28	150	7	3.63 U/mL	The endophytic fungus with the higher protease activity demonstrated total efficacy in the removal of the consolidated biofilm of *S. aureus.*
Meshram and Saxena [27]	India	*Aegle marmelos*	*Lasiodiplodia pseudotheobromae*	ITS-rDNA	ND	26	130	7	6514 U/mL	The endophytic fungus possesses potential in vitro fibrinolytic potential.
Meshram et al. [28]	India	*Cathranthus roseus*	*Xylaria curta*	28s rDNA	ND	26	130	7	34.11 U/mL	Submerged fermentation was used to produce the fibrinolytic enzyme. This protease is a novel metalloprotease possessing dual activity including direct degradation of fibrin(ogen) or by activating the tissue plasminogen.
Meshram et al. [29]	India	*Cathranthus roseus*	*Xylaria curta*	ITS-rDNA 28s rDNA	ND	28	ND	15	103.56 U/mL	The fibrinolytic enzyme xylarinase was produced by solid substrate fermentation using rice chaff medium. The purified metalloprotease showed in vitro thrombolytic activity and no cytotoxic effect.
Noor et al. [30]	Malaysia	*Hibiscus*	*Fusarium* sp.	18S rRNA ^a^	ND	ND	ND	ND	5284 U/mL	Two fibrinolytic enzymes were purified and characterized based only on molecular weight and effect of pH and temperature.
			*Penicillium* *citrinum*	18S rRNA ^a^	ND	ND	ND	ND	2200 U/mL	
Rajput et al. [31]	India	*Cupressus torulosa D. Don*	*Alternaria* *alternata*	Morphology	7	27	ND	2	162 U/mL	The fungus can be industrially exploited for the synthesis of protease and strain improvement studies can be carried out to enhance enzyme production.
Wu et al. [32]	China	*Chrysanthemum*	*Fusarium* sp.	ITS	ND	28	220	6	137,000 U	The fibrinolytic enzyme, named Fu-P, was purified and identified as a chymotrypsin-like serine metalloprotease. May be a potential candidate for thrombolytic therapy or thrombosis prevention.
Zaferanloo et al. [33]	Australia	*Eremophilia longifolia.*	*Alternaria* *alternata*	Morphology ITS	6.5	30	ND	7	69.86 BAEE units/mg	The protease can be applied to cheese making and in milk-clotting where the fermentation conditions are suitable to the activation of protease

^a^ Data presented in earlier studies referenced in the article. ND: No data.

**Table 2:** Summary of purification steps, characterization, and kinetic properties of protease from endophytic fungi.

Author/Year	Purification Method	Microorganisms	Specific Activity	Purification fold	Molecular Weight (kDa)	Ph Optimum	Temperature Optimum (°C)
Elgammal et al. [22]	Partial purification Ethanol fractionation and precipitation	*Aspergillus ochraceus*	384.2 UI/mg	0.11	ND	8	50
El-Khonezy et. al. [34]	Ammonium sulfate precipitation Sephacryl S-200 DEAE-Sepharose CM-Sepharose	*Aspergillus ochraceus*	111,379.5 U/mg protein	15.3	59	8	50
Li et al. [24]	Ammonium sulfate precipitation DEAE-52 column Sephadex G-75 Octyl Sepharose 4 FF hydrophobic column	*Verticillium* sp.	3775 UI/mg	8.1	31	9–10	50–60
Lindstrom and Belanger [25]	Ultrafiltration 30 Kda, Phenylboronate Column Methanol precipitation	*Acremonium typhinum*	710 UI/units/ng	ND	34	10–11	37
Meshram and Saxena [27]	Partial purification Ammonium sulphate precipitation and dialyze	*Lasiodiplodia* *pseudotheobromae*	3.56 U/mg	2.01	80	ND	ND
Meshram et al. [28]	Ammonium sulfate precipitation Q-sepharose anion exchange	*Xylaria curta*	36.67 U/mg	9.19	~33	8	35
Meshram et al. [29]	Ammonium sulphate precipitationSephacryl S-300 column	*Xylaria curta*	9.22 U/mg	8.37	~33	8	35
Noor et al. [30]	Ammonium sulfate precipitation Hi-Prep 26/10 Desalting Column Hi-Trap Benzamidine FF Column	*Fusarium* sp.	246.92 UI/mg	11.2	~34	7	30
Noor et al. [30]	Ammonium sulfate precipitation Hi-Prep 26/10 Desalting Column Hi-Trap Benzamidine FF Column	*Penicilium citrinum*	198.2 UI/mg	9.7	~34	8	40
Wu et al. [32]	Ammonium sulfate precipitation MonoQ Column Superdex 75 Column	*Fusarium* sp.	76,111 UI/mg	158.5	28	8.5	45

ND: No data.

**Table 3:** Risk of bias in individual studies. Fulfilled GRADE criteria.

Author	Study Limitation	Inconsistency	Indirectness	Imprecision	Publication Bias	Overall Quality
Mefteh et al. [20]	√	√	√	√	√	++++
Bhagobaty and Joshi [21]	X	X	√	√	X	+
Elgammal et al. [22]	√	√	√	√	√	+++
El-Khonezy et. al. [34]	√	√	√	√	√	++++
Galeano et al. [23]	√	√	X	√	√	+++
Li et al. [24]	X	X	√	X	√	+++
Lindstrom and Belanger [25]	X	X	√	X	√	+
Matias et al. [26]	√	X	X	√	√	++
Meshram and Saxena [27]	√	X	√	Unclear	√	+++
Meshram et al. [28]	√	X	√	Unclear	√	+++
Meshram et al. [29]	√	X	√	Unclear	√	+++
Noor et al. [30]	√	X	√	X	X	++
Noor et al. [30]	√	X	√	X	X	++
Rajput et al. [31]	√	√	√	Unclear	√	+++
Wu et al. [32]	X	√	√	√	√	++++

Grade Factors: √, No Serious Limitations; X, Serious Limitations; Unclear, Unable to rate item based on available information.; For overall quality of evidence: +very low; ++ low; +++ moderate; ++++ high.

**Figure A1:**
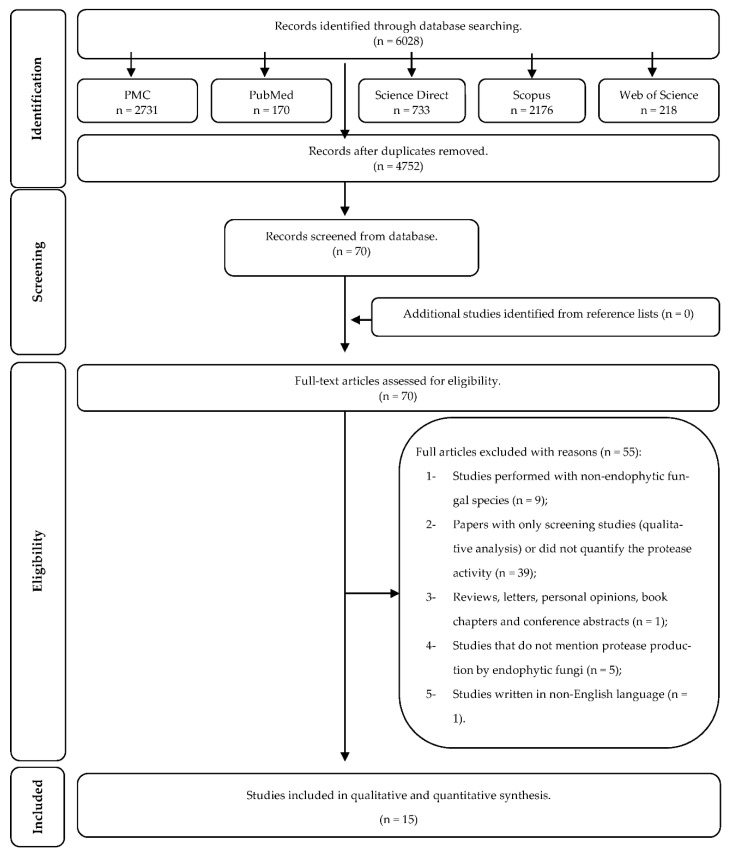
Flow Diagram of literature search and selection criteria adapted from.

## Data Availability

This study did not report any data.

## Appendix A

**Table A1:** Excluded articles and reasons for exclusion (*n* = 55).

References	Reason for Exclusion
Abou El-Kassem et al. [49]	4
Alberto et al. [50]	2
Amobonye et al. [51]	3
Ayob and Simarani [42]	2
Baazeem et al. [43]	2
Bajwa et al. [52]	1
Bastos et al. [53]	4
Bensaci et al. [54]	2
Bezerra et al. [55]	2
Bezerra et al. [56]	2
Borgi et al. [57]	1
Bryant et al. [58]	4
Cairney and Burke [59]	1
da Silva et al. [60]	2
da Silva et al. [61]	1
De Azevedo Silva et al. [62]	2
Devi et al. [63]	2
El-Gendy [64]	1
Fouda et al. [38]	2
George et al. [65]	2
Gupta et al. [36]	2
Hassan [66]	2
Indarmawan et al. [67]	1
Jagannath et al. [68]	2
Jalili et al. [69]	2
Kapoor et al. [70]	2
Katoch et al. [71]	2
Katoch et al. [72]	2
Kudryavtseva et al. [73]	5
Kumar et al. [74]	4
Kuzhalvaymani et al. [75]	1
Leake and Read [76]	1
Lindstrom et al. [77]	2
Liu et al. [78]	1
Lopez-Llorca et al. [79]	4
Lumyong et al. [80]	2
Maccheroni et al. [81]	2
Martins et al. [82]	2
Meshram et al. [39]	2
Mishra et al. [83]	2
Monteiro et al. [84]	2
Orlandelli et al. [85]	2
Prathyusha et al. [86]	2
Rajagopal et al. [87]	2
Rajesh and Ravishankar Rai [88]	2
Reddy et al. [89]	2
Santos et al. [90]	2
Seshagiri and Tallapragada [91]	2
Sharma et al. [92]	2
Silva et al. [93]	2
Sopalun and Iamtham [94]	2
Sopalun et al. [95]	2
Swetha et al. [96]	2
Wu et al. [97]	2
Zaferanloo et al. [98]	2

Reasons for exclusion:

- Studies performed with non-endophytic fungal species (*n* = 9);
- Papers with only screening studies (qualitative analysis) or did not quantify the protease activity (*n* = 39);
- Reviews, letters, personal opinions, book chapters, and conference abstracts (*n* = 1);
- Studies that do not mention protease production by endophytic fungi (*n* = 5); and
- Studies written in non-English language (*n* = 1).

## Appendix C

**Table A2:** Search strategies with appropriated key words and MeSH terms.

Database	Search
PMC (10 September 2021)	((“peptide hydrolases”[MeSH Terms] OR (“peptide”[All Fields] AND “hydrolases”[All Fields]) OR “peptide hydrolases”[All Fields] OR “protease”[All Fields]) OR (“peptide hydrolases”[MeSH Terms] OR (“peptide”[All Fields] AND “hydrolases”[All Fields]) OR “peptide hydrolases”[All Fields] OR (“proteolytic”[All Fields] AND “enzyme”[All Fields]) OR “proteolytic enzyme”[All Fields]) OR (“peptide hydrolases”[MeSH Terms] OR (“peptide”[All Fields] AND “hydrolases”[All Fields]) OR “peptide hydrolases”[All Fields] OR “peptidase”[All Fields]) OR (“peptide hydrolases”[MeSH Terms] OR (“peptide”[All Fields] AND “hydrolases”[All Fields]) OR “peptide hydrolases”[All Fields] OR “proteinase”[All Fields])) AND ((endophytic[All Fields] AND (“fungi”[MeSH Terms] OR “fungi”[All Fields] OR “fungus”[All Fields])) OR (endophytic[All Fields] AND (“microbiology”[Subheading] OR “microbiology”[All Fields] OR “fungi”[All Fields] OR “fungi”[MeSH Terms])) OR mycoendophyte[ All Fields])
PubMed (10 September 2021)	(protease OR proteolytic enzyme OR peptidase OR proteinase) AND (endophytic fungus OR endophytic fungi OR mycoendophyte)
Scopus (10 September 2021)	( ( ( ( ( protease ) OR proteinase ) OR peptidase ) OR proteolytic AND enzyme ) ) AND ( ( ( endophytic AND fungi ) OR endophytic AND fungus ) OR mycoendophytics ) AND ( LIMIT-TO ( DOCTYPE, “ar” ) OR LIMIT-TO ( DOCTYPE, “sh” ) )
Science Direct (10 September 2021)	(protease OR proteolytic enzyme OR peptidase OR proteinase) AND (endophytic fungus OR endophytic fungi OR mycoendophyte)—Limited to: Research articles, Discussion, News, Short communications and Other
Web of Science (10 September 2021)	#1 TS = (protease OR proteolytic enzyme OR peptidase OR proteinase) AND #2 TS = (endophytic fungus OR endophytic fungi OR mycoendophyte) COMBINE #1 and #2
Google Scholar (10 September 2021)	(protease OR proteolytic enzyme OR peptidase OR proteinase) AND (endophytic fungus OR endophytic fungi OR mycoendophyte)
